# Incidence of BK Virus Nephropathy (BKVN) in Renal Transplant Recipients

**Published:** 2012-08-01

**Authors:** A. Pezeshgi, A. Ghods, H. Keivani, M. Asgari, M. Shatty

**Affiliations:** *Hasheminejad Hospital, Tehran University of Medical Sciences, Tehran, Iran*

**Keywords:** Nephropathy, BK virus, JC virus, kidney transplant

## Abstract

Background: BK virus nephropathy (BKVN) is one of the complications of renal transplantation that causes graft loss in renal transplant recipients.

Objective: To determine the incidence of BKVN after renal transplantation in Hasheminejad Hospital, Tehran, Iran.

Methods: In this analytical cross-sectional study, we evaluated 31 consecutive kidney transplant recipients (21 men and 10 women) for BK and JC viral infections and BKVN during one year after transplantation, Urine of patients was tested for the presence of decoy cells and DNA of BK and JC virus by PCR. The serum load of BK and JC virus was assessed in patients 3, 6, 9, and 12 months after transplantation. Renal biopsy was performed in presence of allograft dysfunction or viral load >10^7^ copies/mL.

Results: The prevalences of decoy cells and BK and JC viral DNA in urine of patients were 16%, 29%, and 23%, respectively. BK or JC virus was found in 45% of the urine samples. During one year follow-up, no cases of BKVN was observed.

Conclusion: Despite a high rate of BK viral infection, no one with BKVN was observed in our kidney transplant recipients. Therefore, screening of all transplant recipients for BKVN is not recommended.

## INTRODUCTION

Three *Polyomavirus*—JCV, BKV, and SV40—cause disease in humans who are the natural host for JCV and BKV [1]. These viruses enter the body in childhood [2, 3]. The initial infection may occur through fecal–oral transmission, respiratory tract and through the placenta. They can also be transmitted through organ transplantation [4]. After viremia, **u**rinary epithelium, lymphoid tissue and the brain are infected [5]. Virus activity begins in immunocompromised conditions such as immunosuppressed kidney transplant recipients and HIV positive patients [2, 6, 7]. Although SV40 was isolated from kidney transplant biopsies, its importance has not already been well described [8, 9]. Both BK and JC viruses were identified in 1971, but their significance was limited [4]. With the use of new powerful immunosuppressive therapies, Polyomavirus nephropathy was considered [10, 11]. 

Nephropathy is usually caused by BK virus. Up to 8% of transplant recipients will be affected and often cause graft loss or permanent dysfunction [12]. BK virus nephropathy (BKVN) is asymptomatic and presented as a gradual increase in serum creatinine. The pathological picture of tubulointerstitial nephritis can mimic rejection, however, the treatments for these two conditions are different: While treatment of tubulointerstitial nephritis is by dose reduction of immunosuppressant treatment of rejection is by increase in immunosuppressant dose [4].

BKVN is one of the main causes of graft dysfunction [2-13] and presents with hemorrhagic cystitis, ureteral obstruction and nephritis [2, 6, 7, 13]. BKVN is reported regardless of transplant protocol used. BKVN is particularly common with triple therapy protocol (tacrolimus, mycophenolate mofetil and prednisone) [6]. BKVN is diagnosed by measuring viral DNA load in urine and plasma, or identifying urine decoy cell [3, 13, 14]. The definite diagnosis is made with renal biopsy and immunohistochemistry.

According to the results of previous studies, BKVN results in the loss of allograft in 45% of patients with nephritis. There is no safe and effective antiviral therapy, and physicians have to adopt a prophylactic measure for this disease. Early diagnosis and decreasing the dose of immunosuppressant can prevent the graft loss [6, 7, 13].

So far no study was conducted on the incidence of BKVN among transplant recipients in our country. This study was therefore conducted to examine the incidence of BKVN in transplant recipients in Hasheminejad Hospital, Tehran, Iran.

## PATIENTS AND METHODS

In this analytical cross-sectional study conducted in Transplant Center of Hasheminejad Hospital, Tehran, Iran, 31 (21 men and 10 women) consecutive kidney transplant recipients from living donors were studied. After obtaining informed written consent, the patients were evaluated for the presence of urinary decoy cells, and BK and JC viral DNA by qualitative PCR. Patients were excluded from the study if they were unable to continue due to any causes (*e.g.*, distance), if they were not happy to continue participation in the study, or if they died. We measured BK and JC viral load in plasma of patients with DNAuria at 3, 6, 9, and 12 months of transplantation. Transplant kidney biopsy was performed if serum creatinine increased more than 25% of the baseline (renal dysfunction) or serum viral load exceeded 10^7 ^copies/mL. The data obtained were analyzed by SPSS^®^ ver 17.

## RESULTS

Thirty-one patients (21 men and 10 women) received kidney transplant from living donors. The mean±SD age of patients was 38.3±12.8 (rage: 17–59) years. There was no statistically significant difference between the age of men and women (39.3±12.2 *vs*. 36.3±14.3 years, respectively; p=0.539). After transplantation, patients received prednisone, cyclosporine, and mycophenolate mofetil. The mean±SD doses of drugs at discharge were 30.3±5.8 mg, 308±87.7 mg, and 1.98±0.09 g, respectively. Eleven patients received anti-thymocyte globulin (ATG).

One week after transplantation, five (16%) patients had urinary decoy cells. Urine was found positive for the qualitative BK and JC DNA viruses in nine (29%) and seven (22%) patients, respectively.

There was no significant difference between women and men for positivity of urinary BK or JC virus (OR: 1.3, 95% CI: 0.3–6).

There was no statistically significant difference in the mean doses of immunosuppressant drugs between patients with infection (positive for at least one of three tests) and those without infection.

There was no significant difference in prevalence of BK virus infection between patients received anti-thymocyte globulin and those who did not (p=0.809).

Of 14 patients infected with at least one of the two BK or JC viruses, three died—one committed suicide, and two died of sepsis. 

Sera of 10 patients were tested every three months for a period of one year for measurement of BK and JC viral load by quantitative DNA PCR. Three months after transplantation, the mean±SD serum viral load was 578±102 (range: 0–300) copies/mL. After 6, 9 and 12 months of transplantation no virus was detected. The mean serum creatinine levels measured in these 10 patients every three months was less than 1.2 mg/dL. Only in one patient serum creatinine was increased to more than 25% of the baseline value because of discontinuation of immunosuppressant drugs due to severe sepsis. No case of nephropathy induced by these viruses, was observed in our renal transplant recipients.

## DISCUSSION

Different prevalence ratios of BK virus infection have been reported. In a study from Ahwaz Jondi Shapur University,** t**he prevalence of BK virus infection in allograft recipients before and after kidney transplantation was 6.4% and 12.8% one month after transplantation, and 38.5% after four months of transplantation [15]. In Poland, BK virus DNA in one or more serum samples was found in 19.1% of patients—only one patient developed nephropathy [10]. In another study, the prevalence of BK and JC virus infection in kidney transplant recipients, patients with chronic kidney disease, and a group of healthy subjects, measured by PCR, was 30.5%, 17.6%, and 3.9%, respectively; most of them had BK virus [11]. The prevalence of BK virus infection in USA, assessed by serum DNA PCR and urine decoy cell, was 7.4% [12]. In another study, 120 transplanted patients with allograft dysfunction were assessed; the prevalence of urine decoy cell, viruria, and viremia was 25%, 61.7%, and 42.5%, respectively [16].

It seems that the reported prevalence of infection is influenced by many factors including sensitivity and specificity of the test used, type of sample examined (urine or serum), and the elapsed time after kidney transplantation. Despite the low specificity (about 52%) of urine decoy cells for the diagnosis of active infection, it has a high negative predictive value of 98% [12]. It is as a inexpensive screening test for identifying patients at risk of BK viral infection. Obviously, depending on the clinical circumstances, certain patients at risk may need being tested by other techniques such as PCR and renal biopsy.

In our study, 10 patients were infected with at least one of the two BK or JC viruses during one-year follow-up; no cases of nephropathy induced by these two viruses were observed. 

The prevalence of BKVN in a study on 160 patients, assessed by pathologic evaluation of renal biopsy samples evaluated under light microscopy and immunohistochemistry, was 13.1% [17]. Soleimanian and colleagues, who assessed all kidney biopsies by immunohistochemistry in Shariati Hospital between 2001 and 2006, reported BK virus nephropathy in 0.93% of 108 allograft biopsies and 1.04% of 96 kidney recipients [1]. In USA, BKVN were reported 1.1% of 158 adult, 4.6% of 542 infant, and 3.5% of 173 infant transplant recipients [9, 18]. In England it was 2.1% [8]. An important limitation of our work was the low sample size of the study.

We found no case of BK virus nephropathy which may be due to living donor transplantation and low levels of immunsuppression. The study sample size was low and thus the results may not have external validity.

## References

[1] Stolt A, Sasnauskas K, Koskela P (2003). Seroepidemiology of the human polyomaviruses. J Gen Virol.

[2] Manitpisitkul W, Drachenberg C, Ramos E (2009). Maintenance immunosuppressive agents as risk factors for BK virus nephropathy: a case-control study. Transplantation.

[3] White LH, Casian A, Hilton R (2008). BK virus nephropathy in renal transplant patients in London. Transplantation.

[4] Bohl DL, Brennan DC (2007). BK virus nephropathy and kidney transplantation. Clin J Am Soc Nephrol.

[5] Reploeg MD, Storch GA, Clifford DB (2001). Bk virus: a clinical review. Clin Infect Dis.

[6] Bohl DL, Brennan DC (2007). BK virus nephropathy and kidney transplantation. Clin J Am Soc Nephrol.

[7] Purighalla R, Shapiro R, McCauley J (1995). Virus infection in a kidney allograft diagnosed by needle biopsy. Am J Kidney Dis.

[8] Li RM, Branton MH, Tanawattanacharoen S (2002). Molecular identification of SV40 infection in human subjects and possible association with kidney disease. J Am Soc Nephrol.

[9] Li RM, Branton MH, Tanawattanacharoen S (2002). Molecular identification of SV40 infection in human subjects and possible association with kidney disease. J Am Soc Nephrol.

[10] Purighalla R, Shapiro R, McCauley J, Randhawa P (1995). BK virus infection in a kidney allograft diagnosed by needle biopsy. Am J Kidney Dis.

[11] Binet I, Nickeleit V, Hirsch HH (1999). Polyomavirus disease under new immunosuppressive drugs: a cause of renal graft dysfunction and graft loss. Transplantation.

[12] Hirsch HH, Knowles W, Dickenmann M (2002). Prospective study of polyomavirus type BK replication and nephropathy in renal-transplant recipients. N Engl J Med.

[13] Bernnan DC, Murphy B, Post TW Management of BK virus-induced (polyomavirus-induced) nephropathy in kidney transplantation. UpToDate.

[14] Dall A, Hariharan S (2008). BK virus nephritis after renal transplantation. Clin J Am Soc Nephrol.

[15] Hirsch HH, Brennan DC, Drachenberg CB (2005). Polyomavirus-associated nephropathy in renal transplantation: interdisciplinary analyses and recommendations. Transplantation.

[16] Hogan TF, Borden EC, McBain JA (1980). Human polyomavirus infections with JC virus and BK virus in renal transplant patients. Ann Intern Med.

[17] Gardner SD, MacKenzie EF, Smith C, Porter AA (1984). Prospective study of the human polyomaviruses BK and JC and cytomegalovirus in renal transplant recipients. J Clin Pathol.

[18] Shah KV (2004). Simian virus 40 and human disease. J Infect Dis.

